# Vaccine-Induced Immune Thrombotic Thrombocytopenia: Clinicopathologic Features and New Perspectives on Anti-PF4 Antibody-Mediated Disorders

**DOI:** 10.3390/jcm13041012

**Published:** 2024-02-09

**Authors:** Yi Zhang, Anna-Lise Bissola, Jared Treverton, Michael Hack, Mark Lychacz, Sarah Kwok, Addi Arnold, Ishac Nazy

**Affiliations:** 1 Department of Biochemistry and Biomedical Sciences, McMaster University, Hamilton, ON L8S 4K1, Canada; zhany85@mcmaster.ca (Y.Z.); trevertj@mcmaster.ca (J.T.); hackm@mcmaster.ca (M.H.); kwoks12@mcmaster.ca (S.K.); 2 Michael G. DeGroote Centre for Transfusion Research, McMaster University, Hamilton, ON L8S 4K1, Canada; bissolaa@mcmaster.ca (A.-L.B.); lychacma@mcmaster.ca (M.L.); 3 Department of Medicine, Michael G. DeGroote School of Medicine, McMaster University, Hamilton, ON L8S 4K1, Canada; 4 Department of Pathology and Laboratory Medicine, Western University, London, ON N6A 5A5, Canada; aarnol9@uwo.ca

**Keywords:** vaccine-induced immune thrombotic thrombocytopenia, adenoviral vector-based vaccines, COVID-19, SARS-CoV-2, platelet factor 4, anti-PF4-mediated disorders

## Abstract

Introduction: Vaccine-induced immune thrombotic thrombocytopenia (VITT) is a rare yet severe adverse complication first identified during the global vaccination effort against SARS-CoV-2 infection, predominantly observed following administration of the ChAdOx1-S (Oxford-AstraZeneca) and Ad26.CoV2.S (Johnson & Johnson/Janssen) adenoviral vector-based vaccines. Unlike other anti-platelet factor 4 (PF4) antibody-mediated disorders, such as heparin-induced thrombocytopenia (HIT), VITT arises with the development of platelet-activating anti-PF4 antibodies 4–42 days post-vaccination, typically featuring thrombocytopenia and thrombosis at unusual sites. Aim: To explore the unique properties, pathogenic mechanisms, and long-term persistence of VITT antibodies in patients, in comparison with other anti-PF4 antibody-mediated disorders. Discussion: This review highlights the complexity of VITT as it differs in antibody behavior and clinical presentation from other anti-PF4-mediated disorders, including the high incidence rate of cerebral venous sinus thrombosis (CVST) and the persistence of anti-PF4 antibodies, necessitating a re-evaluation of long-term patient care strategies. The nature of VITT antibodies and the underlying mechanisms triggering their production remain largely unknown. Conclusion: The rise in awareness and subsequent prompt recognition of VITT is paramount in reducing mortality. As vaccination campaigns continue, understanding the role of adenoviral vector-based vaccines in VITT antibody production is crucial, not only for its immediate clinical implications, but also for developing safer vaccines in the future.

## 1. Introduction

Vaccine-induced immune thrombotic thrombocytopenia (VITT) has received significant attention from the global community as efforts to vaccinate against severe acute respiratory syndrome coronavirus 2 (SARS-CoV-2) continue. As such, gaining an in-depth understanding of VITT is not only vital for clinical management but also holds crucial implications for future vaccine developments and furthering our understanding about the pathophysiology of immune-mediated platelet disorders. The purpose of this literature review is to provide a comprehensive overview of the current research surrounding VITT, including its epidemiology, clinical presentation, and management, as well as its pathophysiology and characteristics shared with other anti-PF4 antibody-mediated disorders. By providing an overview of VITT, we seek to identify knowledge gaps in our current understanding and provide an outline for future investigations.

## 2. Incidence Rates of VITT

Over 50 million doses of Ad26.COV2.S and 240.3 million doses of ChAdOx1-S have been administered globally as of April 2022 and August 2021, respectively [1,2]. The World Health Organization has documented a total of 109 cases of thrombosis with thrombocytopenia syndrome, which includes VITT, following vaccination [3]. Of these, 70 were reported in the U.S. (3.7 per million doses), 35 were from Europe (1.7 per million doses), and 2 were from Brazil and South Africa (0.4 and 0.23 cases per million doses, respectively) [3]. The incidence rates of VITT following ChAdOx1-S vary substantially by region: 17.6 cases per million doses in Nordic countries, and 10 cases per million doses in the UK, compared with 0.2 cases per million doses in Asian countries [3]. Despite the suspension of adenoviral vector-based vaccines by numerous countries, including Canada, United States, and multiple European nations [4,5,6], as of September 2022, ChAdOx1-S and Ad26.COV2.S remain in active use as primary immunization options for adults in a considerable number of countries around the world [7]. This ongoing administration involves nine countries in Latin America, eight in Africa and the Middle East, and five in Asia [7].

Initially considered a risk factor for VITT, the higher incidence of VITT in females was later attributed to the demographic bias in early vaccine recipients, who were primarily healthcare professionals—a group that skews towards females in many countries and was given vaccination priority [8]. Subsequent analyses have identified age as a more relevant risk factor of VITT [8]. A meta-analysis of data from 10 countries showed VITT incidence was lowest at 1 in a million people over 65, increased to 1 in 300,000 among those aged 55 to 64, and peaked in the under-55 age group with a range from 1 in 20,000 to 1 in 60,000 [9]. Geographic location may also represent another possible risk factors for developing VITT. Notably, countries in Asia, such as South Korea, exhibit substantially lower incidence rates of VITT than Western countries [10], although 67.2 million doses of ChAdOx1-S have been administered in the European Union in contrast to 20.0 million doses in South Korea as of November 2023 [11]. Further investigations are needed to determine whether this potential relationship truly exist or is merely a reflection of the lower number of vaccinated individuals. Moreover, this observation also introduces the concept that countries with higher data quality tend to report a higher estimated risk of VITT [12], primarily due to the accuracy in reporting VITT cases and estimating the at-risk vaccinated population [12]. It is important to note that, however, our review may also be influenced by these regional variations in data quality, which may affect the generalizability of our analyses. Nevertheless, the variation in VITT incidence rates across countries emphasizes the imperative for ongoing, vigilant monitoring and management of vaccine-associated complications, particularly as countries continue the administration of adenoviral-vector based vaccines [7].

## 3. Clinical Presentation and Management of VITT

The earliest cases of thrombocytopenia and thrombosis in otherwise healthy individuals after receiving a dose of the ChAdOx1-S vaccine were documented in April 2021 by Greinacher et al. [13], Scully et al. [14], and Schultz et al. [15]. The index patient reported by Greinacher et al. [13] was hospitalized 10 days after vaccination and received various treatments, including anticoagulation (enoxaparin), transfusions of red blood cells and platelets, prothrombin complex concentrate and recombinant Factor VIIa [13]. Despite these interventions, this patient passed away 11 days post-vaccination [13]. The clinical resemblance of unexplainable thrombocytopenia and thrombosis to spontaneous heparin-induced thrombocytopenia (spHIT) prompted investigators to test patient serum for antibodies against platelet factor 4 (PF4/CXCL4) [13], a protein extensively studied for its role in the pathogenesis of HIT [16]. This unique clinical presentation [17] led to the first of many reported cases of thrombotic events caused by adenoviral vector-based vaccines, herein known as VITT.

Current evidence suggests that VITT is induced by pathogenic antibodies that mediate platelet activation in the presence of PF4, which is a chemokine released from activated platelets and is involved in normal coagulation and inflammatory processes [18]. VITT predominantly manifests following primary administration of certain replication-deficient adenoviral vector-based COVID-19 vaccines, namely, the ChAdOx1-S (Oxford-AstraZeneca) and Ad26.CoV2.S vaccines (Johnson & Johnson/Janssen) [13,19]. Despite its rarity, studies have also indicated the occurrence of VITT following subsequent booster doses of COVID-19 vaccines [20]. A recent case series by Mendes-de-Almeida et al. [20] documented 11 VITT patients following either the third or fourth vaccine dose of ChAdOx1-S, with an incidence rate of 0.33 per million doses following these later doses, despite none of the patients having received an adenoviral vector-based vaccine but rather Sinovac or BNT162b2 mRNA vaccines as their primary dose [20].

While HIT typically presents with thrombosis in common locations, such as the deep veins of the legs or the lungs, and may lead to arterial thromboses like limb ischemia or stroke [21], it is also associated with a low median platelet count of 60 × 10^9^/L [22]. In comparison, VITT is characterized by arterial and venous thromboembolisms at more unusual sites, including the cerebral venous sinus (cerebral venous sinus thrombosis, CVST) and splanchnic vein (splanchnic vein thrombosis, SVT) [13,14], and also features thrombocytopenia, with a median platelet count of 27–47 × 10^9^/L [15,23]. Notably, CVST is a rare cerebrovascular condition affecting ~3 per 100,000 in the general population, which can be accompanied by intracranial hemorrhaging, severe neurological dysfunction, and a high mortality rate [24,25,26]. Despite its rarity, CVST is found frequently in VITT patients [27] and is associated with a 2.7-fold higher risk of mortality compared with patients presenting without CVST [14].

According to guidelines from the American Society of Hematology, a diagnosis of VITT should be made according to the following criteria: (1) COVID-19 vaccination 4 to 42 days prior to symptom onset; (2) thrombosis (evidence of venous or arterial thrombosis); (3) thrombocytopenia (platelet count below 150 × 10^9^/L); (4) positive for anti-PF4 antibodies in immunoassays; and (5) significantly elevated D-dimer (exceeding 4 times the upper limit of normal) [28]. When 1 or 2 of these criteria are unmet, “probable VITT” and “possible VITT” are ascribed, respectively [28].

Advances in early diagnosis and enhanced treatment strategies have contributed to a notable reduction in the mortality rate of VITT, decreasing from 47% to 22% in cases with symptom onset after 28 March 2021, since the initial identification of VITT [29]. Moreover, due to its clinical similarities, insights into managing HIT have been instructive for the treatment of VITT [17,30]. Administration of high-dose intravenous immunoglobulin (IVIG) at 1 g/kg daily for two days along with non-heparin-based anticoagulants is recommended for VITT management [17,28]. Although heparin is known to promote platelet activation in HIT, therapeutic doses of heparin have been found to inhibit VITT serum-induced platelet activation in vitro [17,31,32,33]. However, a clinical study involving 220 VITT patients indicated higher mortality rates among those treated with unfractionated heparin (20%) as compared with those administered non-heparin-based anticoagulants (16%) [23]. Platelet transfusions should be avoided due to the elevated risk of thrombosis, unless necessary in situations such as when life-threatening bleeding occurs [28]. For refractory cases of VITT, plasma exchange, corticosteroids, and monoclonal antibodies targeting CD20 on B-cells and complement protein C5 have been utilized, as reviewed in detail by Nadia et al. [31].

## 4. Mechanisms of VITT Pathogenesis

As summarized in Table 1, VITT exhibits pathophysiological similarities to HIT, as both are pro-thrombotic disorders characterized by a pronounced decline in platelet count (relative to baseline platelet levels) and an elevated risk of thrombosis [16,23]. HIT is primarily attributed to the formation of antibodies directed against PF4 complexed with heparin (PF4/heparin), with symptom onset 5 to 10 days after initial heparin administration [16]. Conversely, VITT generally presents 4 to 42 days following the primary dose of adenoviral vector-based COVID-19 vaccine, with anti-PF4 specific antibodies directed against the heparin-binding site [18,28,34]. Given that symptoms in VITT can present as early as four days following vaccination, which is a timeframe insufficient for antibody class switching to IgG to take place, the rapid IgG-specific response seems to suggest a secondary immune reaction, a pattern also observed in HIT [28,35]. However, the onset time of VITT may be less precise compared with HIT due to the reliance on self-reporting upon symptom development for VITT patients, as opposed to the routine monitoring conducted in hospital settings post-heparin administrations in HIT patients.

The prevailing theories behind VITT antibody development are based on immunogen formation caused by PF4 interacting with polyanionic vaccine constituents or being induced by vaccination itself, drawing parallels with HIT pathogenesis. Several studies have demonstrated complex formation between PF4 and ChAdOx1-S, including free hexon proteins, and have been observed to associate with VITT anti-PF4 antibodies, supporting this proposed mechanism [36,37,38]. However, a study by Michalik et al. [38] indicates that while an interaction was observed with PF4 and the vaccine preparation of ChAdOx1-S, PF4 did not show binding to the purified virions of either ChAdOx1-S or Ad26.COV2.S [38]. Considering that ChAdOx1-S contains an up to three-fold higher level of unassembled viral proteins than Ad26.COV2.S, the reactivity of PF4 may be associated with free hexon components rather than assembled virions [38]. It has been hypothesized that upon vaccine injection, immunogenic PF4/polyanionic complexes may form and circulate systemically, engaging monocytes and neutrophils via FcγIIa receptor-dependent mechanisms [39,40]. Subsequently, this activation could drive the differentiation of anti-PF4 B-cells into plasma cells, a process proposed to be facilitated by a prior B-cell pre-priming event resulting from previous exposures to polyanionic components of bacterial or viruses [37,41]. The formation of spHIT antibodies is believed to follow a similar process, although the seroconversion mechanism is not yet fully understood, highlighting a subject of ongoing investigation [42].

Complex formation between PF4 with vaccine excipients was also suggested to induce formation of neoepitopes and anti-PF4 antibody, which include extracellular nucleic acids and proteins from human cell lines used for propagating the adenoviruses during production [43]. Proteomic analysis via liquid chromatography-mass spectrometry and subsequent bioinformatics assessments conducted by Krutzke et al. revealed substantial levels of human origin proteins in ChAdOx1-S (44.3−70.8%) compared with Ad26.COV2.S (0.5%), perhaps explaining the different incident rates associated with the two vaccines [44]. Although aggregate formation was significant between PF4 and ChAdOx1-S [38], Michalik et al. found no significant aggregation between PF4 and Ad26.COV2.S, despite its implication in VITT [38]. Alternatively, Nicolai et al. suggested VITT could arise from unintended intravenous vaccine injection, as they demonstrated in mice that such administration of ChAdOx1-S leads to direct adenovirus to platelet interaction and activation, resulting in thrombocytopenia [45]. However, Gresele et al. argues that the viral load of COVID-19 adenoviral vector-based vaccines is not sufficient to cause coagulation in non-human primates, even if its entire content were to leak into the bloodstream [46]. Concerns have also been raised regarding the possibility of VITT antibody cross-reactivity due to sequence homologies between the SARS-CoV-2 spike protein and two consecutive epitopes on PF4 intrinsic to its heparin-binding region [18,47]. Despite these homologies, Greinacher et al. [47] identified distinct immune pathways for the production of post-vaccination anti-spike antibodies and VITT-related anti-PF4 antibodies as their investigation found no interaction between the spike protein and VITT antibodies, thereby eliminating concerns about their cross-reactivity [47].

Beyond the initial role of neoepitope formation, studies also suggest the contribution of certain vaccine excipients in facilitating proinflammatory responses and promoting site-specific thrombosis. For instance, the presence of ATPase activity in ChAdOx1-S, which could be due to the considerable amounts of heat shock proteins and chaperones, may also induce localized ADP production and subsequent platelet activation at the vaccine injection site [44]. EDTA found in ChAdOx1-S has also been shown to induce vascular hyperpermeability [36,38], and the non-ionic surfactant polysorbate 80 found in both adenoviral vector-based vaccines has the capability to traverse the blood–brain barrier when complexed with nanoparticles [48]. Subsequent to gaining access to the brain, soluble spike protein variants, produced by alternative splicing from DNA-based vaccines, may have an increased residence time at this site due to a lack of dural venous sinuses valves [49]. This extended presence might increase their likelihood of binding endothelial cells expressing angiotensin-converting enzyme 2, thereby elevating the risk of thrombi development in the cerebral sinuses [49] and potentially contributing to the overall immune responses observed in VITT. However, Eichinger et al. [50] noted that VITT onset occurs at a time when the adenovirus and vaccine excipients are likely absent from circulation, making these unlikely contributors to the disorder [50].

Alternatively, recombinant monoclonal VITT antibodies reverse-engineered using mass spectrometry have been helpful in expanding our mechanistic understanding of VITT [51]. Sequence analysis revealed a high concentration of acidic/negatively charged amino acids in the VITT paratope (antibody region involved in antigen binding), facilitating interactions with the heparin binding site on PF4 driven by electrostatic forces [51]. This electrostatic nature of epitope–paratope interactions plus the presence of an equatorial ring of cationic charges on PF4 allow one molecule of PF4 to link multiple VITT antibodies together, forming pathogenic complexes capable of inducing platelet activation without requiring a polyanionic scaffold [51]. While this behavior deviates from the well-documented dependency of classical HIT antibodies on heparin, a similar model has been proposed for the atypical presentations of HIT (such as spHIT) [16,52]. From these findings, Ivanov et al. [51] questioned the involvement of PF4 in initially triggering the formation of VITT antibodies, or whether it may merely contribute to the subsequent thrombi generation, potentially following a vaccine-induced increase in PF4 levels [51]. Despite being oligoclonal, the charge-dependent binding behavior of these antibodies indicates a broad specificity, implying that they may associate with rather than specifically recognize PF4 as the antigen, whereas their initial target might still remain unidentified [51].

## 5. Characterization of VITT Antibodies

Central to gaining an understanding of the functional dynamics and pathophysiological roles in this rare condition is the characterization of these pathogenic anti-PF4 antibodies. Epitope specificity studies conducted by Huynh et al. have identified a restricted epitope of eight surface residues on PF4 critical for the binding of VITT antibodies, suggesting a limited B cell clonality in VITT [18]. Incorporating mass spectrometry analyses, Kanack et al. showed that anti-PF4 antibodies display either a monoclonal or oligoclonal profile [53], standing in contrast to the polyclonal nature of antibodies associated with HIT, thus highlighting the distinct immunological profile of VITT [54]. Building upon these findings, Huynh et al. [18,34] have further revealed two distinct patterns of VITT antibody binding, potentially indicative of a two-site targeting on PF4 [18,34]. The first involves antibodies binding to residues within the well-known heparin-binding site of PF4 [5] and demonstrates PF4-dependent binding [34], while the second shows antibodies engaging additional residues recognized by HIT antibodies, thus demonstrating PF4-indepentent binding [34]. This observation indicates immune response heterogeneity in VITT patients, with variability in antibody binding to PF4 possibly influenced by genetic or physiological factors. However, the mechanisms underlying the development of one-site versus two-site binding are yet to be determined [34].

To further investigate the molecular profile of VITT antibodies, two complementary studies employed mass spectrometry to sequence the variable region of VITT antibodies, with Wang et al. [52] analyzing the samples from five Australian VITT patients and Ivanov et al. [37] examining those from a Canadian VITT patient. Focusing on the third complementarity-determining regions (CDR3), which are known for embedding high levels of diversity from somatic hypermutation and are shaped by the unique immune histories of each individual, both groups identified a pattern of consensus sequence within the heavy chains of the CDR3 of these antibodies across six patients [51,55]. While the exact mutation may vary among individuals, the presence of this consensus sequence suggests the selection of specific clonotypes across various individuals in response to an immunogenic stimulus [51,55]. Furthermore, both studies consistently identified that the lambda light chain in VITT antibodies from all six patients was encoded by the same allele, pointing to a uniform antigen-binding specificity [51,55]. The sequence and structure uniformity likely contribute to the development of these pathogenic antibodies, implying a common immune response pathway consistently inducing a similar immune response amongst VITT patients, potentially indicative of a genetic predisposition [51,55]. Moreover, the epitope of reverse-engineered recombinant VITT antibodies from the five Australian patients [56] overlaps with the heparin-binding site, aligning with analyses conducted by Huynh et al. on antibodies isolated from VITT patient sera [18]. These studies highlight the importance of recombinant VITT antibodies with oligoclonal binding profiles as tools to investigate the underlying genetic makeup of clinical manifestations in these patients [34].

## 6. Drivers of Thrombosis in VITT

Although the precise mechanism of thrombi formation in VITT remains unclear, recent evidence suggests that it may involve activated neutrophils via a process known as NETosis [57]. Upon activation, neutrophils release decondensed DNA coated with histones and various bactericidal proteins, forming structures known as neutrophil extracellular traps (NETs) [58]. NETs serve an immunological role in capturing and destroying pathogens but have also been implicated in the development of thrombosis [58]. In fact, the role of neutrophils and thrombogenicity of NETs has been well characterized in HIT. Previous reports have shown that HIT antibody immune complexes can directly engage with FcγRIIa receptors on neutrophils, triggering their activation and the release of NETs [40,59]. Indirect pathways to NETosis in HIT can also be initiated through platelet-neutrophil interactions mediated by P-selectin and P-selectin glycoprotein ligand-1 [40,59,60]. Consequently, NETs play a pivotal role in thrombi development by both trapping platelets within growing thrombi and by facilitating fibrin deposition [40,59]. Recent studies have demonstrated a consistent elevation in markers characteristic of NETosis in VITT patient sera, including citrullinated histone H3, myeloperoxidase, and cell-free DNA, highlighting the critical role of NETs in thrombi formation [36,57]. As seen with HIT antibodies, VITT antibodies have also been shown to trigger neutrophil activation via FcγRIIa receptors, suggesting a similar underlying process of thrombi development in VITT [57,61].

Perhaps most importantly, recent evidence has implicated NETosis in both the development and exacerbation of CVST in VITT patients. In a study by Jin et al. [26] a high concentration of NETs were found both the plasma and thrombi of CVST patients through examination of CVST tissue sections and quantifying plasma NETosis markers [26]. These NETs not only contribute to thrombi formation, but were also shown to compromise the endothelial barrier and induce a procoagulant state in endothelial cells, possibly further intensifying the CVST pathogenesis [26]. Moreover, CVST patients were found to have enhanced platelet–neutrophil aggregation at thrombus sites, similar to what is seen in HIT [26]. Released PF4 from activated platelets can subsequently induce NETosis through activating neutrophil autophagy pathways in a dose-dependent manner [26], which may hold further implications for anti-PF4 disorders such as VITT. Subsequent studies support these findings by demonstrating elevated NETosis markers in tissue sections obtained from VITT patients who developed CVST [36,62].

Aside from the involvement of neutrophils and NETosis, there may be other factors contributing to CVST development in VITT. Expanding on the earlier-mentioned binding patterns of VITT antibodies on PF4, Huynh et al. [34] found that PF4-independent antibodies, which exhibit a two-site binding profile, have a stronger association with the occurrence of CVST compared with PF4-dependent antibodies, which exhibit a single-site binding affinity [34]. This distinction is evident in the CVST incidence rate of 50.0% in VITT patients whose sera contained both PF4-dependent and -independent antibodies, compared with 5.9% in those whose sera contained only PF4-dependent antibodies [34]. Based on both cellular and humoral findings, a more comprehensive understanding of CVST in VITT, one that encompasses pan-cellular activation, systemic inflammation, extensive neutrophil priming and activation, and various other novel mechanisms such as the potential role of antibody clonality and epitope specificity on PF4 in exacerbating NETosis may better explain the frequency of CVST development in VITT. Table 2 presents a summary of the proposed mechanisms behind VITT antibody production and related pathogenic proinflammatory and thrombotic events. 

## 7. VITT-Mimicking Anti-PF4 Antibodies in HIT and Other Disorders

As mentioned earlier in this review, evidence suggests a degree of resemblance between anti-PF4 antibodies in VITT and those identified in patients with atypical presentations of HIT, such as spHIT [52]. Despite the distinct antibody profiles and clinical presentations associated with HIT, it is a disorder involving anti-PF4 IgG antibodies that activate platelets by engaging FcγRIIa receptors and bind to residues in the heparin-binding region on PF4, a site known to be recognized by VITT antibodies [18,63,64]. The occurrence of spHIT is often observed post-infection or total knee arthroplasty without proximate exposure to heparin and is associated with a high frequency of CVST [65]. Warkentin et al. have recently demonstrated that HIT antibodies exhibit an analogous reactivity profile to VITT antibodies in solid-phase laboratory diagnostic assays, while displaying a degree of variable reactivities in the recognitions of PF4 alone versus PF4/heparin complexes [66]. However, in fluid-phase EIAs where complexes of anti-PF4 IgG and PF4 with or without heparin are pre-formed before immobilization, spHIT antibodies exhibited a heparin-enhanced binding behavior, while VITT antibodies display a significant heparin-inhibitory behavior, potentially elucidating the laboratory and clinical differences between these disorders [66].

VITT-mimicking anti-PF4 antibodies were also found in two patients with monoclonal gammopathy of undetermined significance (MGUS), which is a neoplastic condition thought to be related to abnormal growth and proliferation of plasma cells in the bone marrow [67]. In both reported patients, the MGUS antibodies were produced from a single clone of anti-PF4 plasma cells, and resulted in recurrent thrombosis and thrombocytopenia despite them having no previous exposure to heparin [68,69]. However, the case reported by Kanack et al. [69] may represent an even more complex scenario: the patient received an initial dose of Ad26.COV2.S followed by two doses of BNT162b2 mRNA vaccines [69]. Subsequent treatment via intravenous heparin administration led to a significant drop in platelet counts, though the timing of heparin administration relative to the vaccinations was not indicated [69]. While no adverse effects were observed subsequent to these vaccinations, the precise impact of the adenoviral vector-based vaccine on the condition of the patient remains undetermined [69]. On the other hand, the patient documented by Greinacher et al. [68] had not received any COVID-19 vaccines at the time of reporting nor been given heparin in subsequent treatments, suggesting their symptoms could be attributed to pre-existing underlying conditions [68].

Although no strong genomic link is associated with the development of HIT, it may be possible that VITT is a result of genetic predisposition triggered by the administration of virus-based vaccines. For instance, three cases of VITT have been reported following vaccination with the mRNA-1273 (Moderna) vaccine, and another with the virus-like particle vaccine for nine-valent HPV (Gardasil-9) [70,71]. The latter patient presented with platelet-activating anti-PF4 antibodies without history of thrombosis or exposure to heparin, nor had she received any COVID-19 vaccines [71]. A concurrent HPV infection, however, may have contributed to the formation of PF4/polyanionic complexes [71]. As such, anti-PF4 antibody-mediated hypercoagulability may also go beyond heparin and vaccine exposure. Warkentin et al. [64]. recently reported two patients presenting with a VITT-like anti-PF4 disorder associated with adenovirus infection [64]. Both patients tested positive for adenovirus infection via nasopharyngeal swabs; one patient received two doses of Moderna COVID-19 vaccines 15 months earlier, while the other patient experienced a mild case of SARS-CoV-2 infection 16 months prior [64]. Serum samples from these two patients were positive for anti-PF4 antibodies in both solid and fluid phases assays, and in PF4-enhanced platelet-activation assays, and demonstrated binding to the heparin-binding region on PF4 [64]. A similar finding was also reported by Schönborn et al. [72], who identified nine patients with VITT-like clinical and humoral profiles from a retrospective analysis of repository serum samples dated before 2020 [72]. These individuals experienced acute thrombocytopenia, significantly elevated D-dimer, and severe thrombotic events, including arterial strokes and CVST, without recent exposure to heparin or adenoviral vector-based vaccines [72]. Thus, the association of pro-thrombotic anti-PF4 antibodies with disorders showing clinical and/or pathological similarities to VITT may be indicative of an unknown trigger for pathogenic antibody production beyond our current scope of understanding.

## 8. VITT Antibody Persistence and Clinical Consequences

While the acute clinical manifestations demand urgent attention, it is also critical to examine the longitudinal characteristics of VITT anti-PF4 antibodies, which could provide insights on long-term patient management strategies. Comparison with HIT often offers an invaluable perspective for the understanding of VITT due their clinical similarities. Unlike classical immunological memory, which leads to long-lasting heightened immunity upon re-exposure to an antigen, the development of anti-PF4/heparin antibodies in HIT is typically transient, only lasting a median of 50–80 days [73,74], and their ability to activate platelets is lost before they become undetectable in serum [75]. HIT patients generally have no observed immune response following subsequent heparin treatments, suggesting the lack of immune memory from a clinical perspective [74,76]. The consensus recommendation suggests that for patients with a history of HIT who no longer have circulating platelet-activating anti-PF4/heparin antibodies, it is advisable to use heparin during surgical procedures and switch to an alternative anticoagulant post-operatively [77]. Interestingly, while HIT patients exhibit a higher propensity (8/17, 47%) for re-developing platelet-activating anti-PF4/heparin antibodies upon subsequent intra-operative heparin re-exposure, subsequent non-surgical heparin treatment does not appear to cause antibody redevelopment [77]. Warkentin et al. [77] report only one patient (1/17; 5%) who developed recurrent HIT (heparin-independent platelet-activating HIT antibodies; thrombosis and thrombocytopenia) following intra-operative re-exposure to heparin [77]. Despite the higher-than-expected rate of seroconversion in patients with a history of HIT, the redevelopment of HIT is relatively rare [77].

Like HIT antibodies, VITT antibodies lose their ability to activate platelets before becoming undetectable in serum [78,79]. However, preliminary evidence suggests that VITT may involve a more durable antibody response, potentially leading to prolonged clinical symptoms [80,81,82]. In a 6-month study conducted by Kanack et al. [82], nine patients with VITT following Ad26.COV2.S vaccination showed no subsequent thrombosis and 78% did not experience thrombocytopenia after the acute phase [82]. Similarly, in a longitudinal study consisting of 71 VITT patients, Schönborn et al. [80] found that platelet-activating anti-PF4 antibodies became undetectable in 87% of patients over a mean of 79 weeks following initial symptom onset, with no further episodes of thrombosis or thrombocytopenia observed in 93% of patients, suggesting a generally low risk of VITT recurrence after the initial stage of symptom onset [80].

Antibody persistence was observed in a subset of patients. A case report by Roberge and Carrier describes a VITT patient who continued to exhibit persistent thrombocytopenia and tested positive for platelet-activating antibodies for more than 18 months following the initial VITT diagnosis [81]. Kanack et al. also observed seven patients with antibodies persisting after 181 days; however, only one exhibited platelet-activating properties, and two experienced mild thrombocytopenia recurrence related to VITT [82]. Schönborn et al. [80] found half (39/71; 55%) of all VITT patients exhibited EIA-detectable anti-PF4 antibodies and 6/71 (8.5%) continued to produce platelet-activating anti-PF4 antibodies for greater than 18 months [80]. Moreover, the authors noted 2/71 patients (2.8%) with recurrent thrombocytopenia and 2/71 patients (2.8%) with recurrent thrombosis [80]. Patients with thrombocytopenia alone were receiving proper anticoagulation, while patients with thrombosis alone were not properly anticoagulated [80]. Only 1/71 (1.4%) patient in this study experienced recurrent thrombocytopenia and thrombosis despite anticoagulation, and this patient also tested positive for platelet-activating anti-PF4 antibodies, likely representing a case of persisting VITT [80].

It remains unclear why some VITT patients exhibit persistent symptoms while others do not. One hypothesis is the involvement of long-lived plasma B cells and/or the development of immune memory, which could contribute to this persistence, in contrast with the observations made in HIT [76], although this remains speculative. As such, longitudinal follow-up and consistent anticoagulation for VITT patients remains of utmost importance. Along with the patient reported by Roberge and Carrier, 7/9 (77.8%) patients followed by Kanack et al. and 28/64 (43.8%) patients followed by Schönborn et al. remained on anticoagulants during the study period to mitigate long-term symptoms [80,81,82]. While anticoagulation is effective in most cases, evidence suggests some VITT patients may be at risk of persistent and recurrent thrombocytopenia and/or thrombosis [80,81,82]. Based on these studies, there appear to be two antibody profiles of VITT patients: (1) those whose platelet-activating antibody diminishes over time, and (2) those with persistent platelet-activating antibodies. However, the exact duration of anti-PF4 antibody persistence is largely unknown due to the limited number of longitudinal studies and patient samples available. Moreover, drastic variation among individuals, both within and across various longitudinal studies, suggests that there may be differences in immune responses between patients [80,81,82]. Nevertheless, these observations underscore the necessity to investigate the underlying causes of this persistence and emphasize the importance of developing targeted long-term monitoring and management strategies for VITT patients.

## 9. Conclusions

The widespread use of adenoviral vector-based COVID-19 vaccines revealed VITT as a rare, yet significant adverse effect associated with this vaccine delivery platform. The occurrence of VITT underscores the challenges of monitoring and managing adverse events on a large scale, thus emphasizing the importance of continuous surveillance and improvement in vaccination strategies. Collaborative efforts within the scientific community are crucial for developing guidelines to ensure safety during vaccine formulation, testing, and administration. Further research beyond preliminary studies is, therefore, required to provide evidence of the involvement of adenoviral-vector or non-viral constituents and confirm these proposed mechanisms. The sequence of events leading to VITT, the reasons behind the differences in incidence rates of ChAdOx1-S and Ad26.CoV2.S, whether some individuals are predisposed to develop VITT IgG antibodies, the persistence of VITT antibodies, and the prevalence of thrombi at specific sites, such as CVST, are questions that remain unanswered. Additionally, the identification of various VITT-like syndromes caused by antibodies in the absence of heparin or vaccine exposure represents a key area of future research that may provide us with novel insights into other anti-PF4 antibody-mediated disorders.

**Table 1:** A clinical and immunological overview of HIT and VITT.

	HIT	VITT
Pathophysiology	Triggered by antibodies against PF4-heparin complexes [16]	Triggered by anti-PF4 specific antibodies, post vaccination [28]
Symptom onset	Typically 5–10 days after heparin administration [16]	Typically 4–42 days after adenovirus vector-based COVID-19 vaccine administration [28]
Clinical presentation	Thrombocytopenia, elevated risk of thrombosis [16]	Thrombocytopenia, high frequency of thrombosis at atypical sites like arterial and cerebral venous sinus [65]
Antibody characteristics and persistence	Transient anti-PF4/heparin antibodies, median duration of 50–80 days [73,74]	Persistent anti-PF4 antibodies, some cases exceeding 18 months [80,81]
Recurrence	Higher propensity for re-developing anti-PF4/heparin antibodies upon intra-operative heparin re-exposure. However, redevelopment of HIT is rare [74,76,77]	A subset of patients shows persistent thrombocytopenia and platelet-activating antibodies, with some cases of recurrent thrombosis and thrombocytopenia [80,82]
Recommended treatment	Non-heparin anticoagulant [42]	IVIG [17]

**Table 2:** Proposed mechanism of VITT pathogenesis.

	Mechanism	Literature
VITT antibody formation	The formation of neoantigens by adenovirus capsid hexon proteins or vaccine excipients (protein impurities or extracellular DNA) binding to PF4 triggers VITT antibodies production by anti-PF4 B cells, although PF4 binding was not observed with purified vaccine virions.	Greinacher et al. Baker et al. Michalik et al. [36,37,38]
	Direct platelet activation and thrombocytopenia were observed in mice following intravenous injection of ChAdOx1-S. However, even if all the vaccine contents spill over into the bloodstream, the viral load of COVID-19 adenoviral vector-based vaccines is unlikely to trigger such a response.	Nicolai et al. Azzarone et al. [45,83]
Proinflammatory and thrombotic events contributing to pathogenesis	ChAdOx1-S contains substantial amounts of human cell line impurities, including heat-shock proteins, that may mediate platelet activation at the injection site.	Krutzke et al. [44]
	DNA-based COVID-19 vaccines can lead to the production of soluble spike protein variants via splicing events. Due to the absence of dural venous sinus valves, prolonged exposure to spike protein variants may contribute to the development of thrombi in the cerebral sinuses.	Kowarz et al. [49]
	ChAdOx1-S stabilized with EDTA may increase vascular permeability and cause dermal vessel leakage, enhancing the spread of proinflammatory factors. Additionally, the surfactant polysorbate 80 in both ChAdOx1-S and Ad26.CoV2.S can cross the blood–brain barrier and enter brain endothelial cells when complexed with nanoparticles, possibly localizing thrombosis to the cerebral sinuses. However, the replication-deficient adenovirus and other vaccine excipients are unlikely to be still circulating given the timing of VITT symptom onset.	Greinacher et al. Choi et al. Kowarz et al. Eichinger et al. [36,48,49,50]
	VITT antibodies were shown to activate neutrophils, leading to NETosis, which is the major driver of thrombosis in VITT, but does not significantly contribute to thrombocytopenia. NETosis has also been implicated in CVST, potentially influencing its prevalence in VITT, although direct evidence of this connection is limited.	Jin et al. Greinacher et al. Leung et al. [26,36,57]
